# Emergence of *Vibrio* and related genera infections in a hotspot of climate risks, southern Spain, 2010–2023

**DOI:** 10.1016/j.onehlt.2025.101267

**Published:** 2025-11-06

**Authors:** Sandra Tapia-Poza, Estefanía Jurado-Tarifa, Inmaculada Guerrero-Lozano, Teresa Trujillo-Soto, Fátima Galán-Sánchez, Juan M. Sánchez-Calvo, Joaquín A. Triñanes-Fernández, Jaime Martínez-Urtaza, Manuel A. Rodríguez-Iglesias

**Affiliations:** a Biomedical and Innovation Research Institute of Cádiz (INiBICA), Cádiz, Spain; b Puerta del Mar University Hospital, Cádiz, Spain; c University of Cádiz, Cádiz, Spain; d Jerez University Hospital, Jerez de la Frontera, Cádiz, Spain; e University of Santiago de Compostela, Santiago de Compostela, Spain; f Universitat Autònoma de Barcelona, Barcelona, Spain

**Keywords:** Vibrio, Shewanella, Clinical infections, Surveillance, Water warming, One health

## Abstract

*Vibrio* has emerged as one of responsive pathogens to the conditions imposed by global warming, with an increasing impact in northern Europe. The epidemiological significance of these pathogens remains undetermined in the south of Europe. We analyzed clinical and epidemiological data from cases in southern Spain, a well-established climate hotspot. A 13-year retrospective analysis identified 167 clinical infection cases. Monthly seawater temperatures since 2010 were also evaluated to assess their correlation with infection occurrence. *V. alginolyticus*, *Shewanella putrefaciens*, *S. algae*, *V. parahaemolyticus*, *V. fluvialis*, and *V. vulnificus* were the most frequently isolated species from clinical cases such as otitis, gastroenteritis, infected wounds, and sepsis. An increase in incidence was observed over the study period in parallel to the warming trend in coastal waters. While this suggests an association between environmental change and *Vibrio* infections, additional factors such as population exposure patterns may likely also contribute. These findings underscore the need for surveillance systems to monitor the burden of these infections.

## 1 Introduction

There is mounting evidence to support that climate change is already threatening human health [1]. Heat waves, meteorological disasters, drought, or glacial melting are becoming more frequent with direct and indirect effects on several elements directly linked to human health, including the decline in water supplies, crop loss, and changes in distribution patterns of disease vectors and exacerbating epidemics [2]. These effects are even more relevant in those climate-sensitive pathogens whose growth and infection profiles are closely connected with environmental variations, such as changes in temperature or water availability [3]. Thus, climate change is reshaping social behaviors, increasing exposure to risks and altering disease epidemiology. A representative case of that is *Vibrio* spp. [4]. These gram-negative bacteria that inhabit marine or estuarine environments, and together with *Shewanella*, cause a variety of human infections, including gastroenteritis, wounds and ear infections, and septicemia [5]. The distribution and abundance of these bacteria correlate with water salinity and Sea Surface Temperatures (SSTs) [6,7] which has been subjected to an unstoppable warming trend in recent decades, showing record levels in recent years. *Vibrio* infections have steadily increased responding to warming, spreading to areas previously unreported for these pathogens [8]. The epidemiological expansion of *Vibrio* infections has been parallel to the increase of areas with ecological suitability for these organisms, with a clear pattern of northward shift reaching areas close to the arctic circle [9]. This new ecological context for *Vibrio* has led to more frequent infections in northern Europe, particularly the Baltic Sea, where moderate salinities create favorable conditions for the bacteria [7]. The implementation of a comprehensive surveillance system in these countries, including vibriosis and its pathogens as notifiable, has improved case identification and provided a clearer view of *Vibrio* infections in northern Europe, which have increased in recent decades, particularly during heatwaves and warm coastal waters [10].

Despite the growing relevance of *Vibrio* infections in the north of Europe [11], the epidemiology of these diseases in the southern regions has remained largely undetermined. The high salinity prevailing around the Mediterranean Sea and Atlantic coasts are considered adverse conditions for *Vibrio,* making the presence of these organisms unlikely despite the warm conditions [12]. However, some recent developments suggest that, at least in some areas, the presence of *Vibrio* in these regions is not so rare as assumed initially. The more frequent extreme precipitation events around the Mediterranean region are contributing to the influx of fresh water into the sea, with a subsequent reduction of salinity and, along with the warm temperatures, may provide suitable conditions to boost *Vibrio* populations [13]. Recent *Vibrio* cases, including some fatalities, in northern areas of Italy associated with flooding events [14] have been related to this pattern, raising the alarm and the need to introduce monitoring systems in areas of risk.

The south of Spain, a climate risk hotspot located a few km away from Africa, is heavily impacted by extreme heat [15], mosquito-borne diseases [16], and other vector-borne illnesses [17]. However, research on the effects of climate change on waterborne infections is lacking, and the epidemiological status and risks of these diseases in the region still remain undetermined.

This study aimed to identify *Vibrio* and related infections along the Atlantic coast of Cádiz (southern Spain), analyzing locally acquired cases to provide an overview of their occurrence and associated environmental patterns over a 13-year period. Clinical strains were sequenced to examine genetic profiles, virulence factors, and antibiotic resistance genes, while evaluating the link between warming coastal waters and the incidence of *Vibrio* infections.

## 2 Methods

### 2.1 Data sources

A retrospective study from 2010 to 2023 examined clinical records on *Vibrio* spp. and related genera (Shewanella and Photobacterium) in Cádiz (southern Spain). Clinical records and microbiological reports were retrieved from two institutions within the Atlantic region of Cádiz, including Jerez University Hospital and the reference hospital Puerta del Mar University Hospital. Patient data, including sex, age, diagnosis, comorbidities, and infection source, were collected. SST data from NOAA's Geo-Polar Blended SST Analysis were used to assess its correlation with infections near Bahia de Cadiz [18].

### 2.2 Isolation and identification

Clinical samples were cultured using routine methods, and bacterial isolates were identified biochemically with MicroScan WalkAway (Beckman Coulter, Brea, USA). In 2014, microbial identification by matrix-assisted laser desorption ionization/time-of-flight mass spectrometry (MALDI-TOF) fingerprinting using Biotyper (Bruker Daltonics, Bremen, Germany) was incorporated in the diagnostic routine. Antimicrobial susceptibility testing (AST) was performed in some cases using standard disk or gradient diffusion methods, following Clinical & Laboratory Standards Institute (CLSI) or European Committee on Antimicrobial Susceptibility Testing (EUCAST) criteria applicable at the time [19]. However, because interpretive standards changed over the 13-year period and results were not systematically available for all isolates, phenotypic AST data were not included in this retrospective analysis. Instead, resistance profiles were inferred from whole-genome sequencing data.

### 2.3 Whole genome sequencing

From the total pool of isolates identified, 59 *Vibrio* and *Shewanella* strains were selected for whole-genome sequencing. Selection criteria included strain viability, species distribution across the study period, and representation of different clinical sources. This strategy was aimed at capturing the genetic diversity of the main species observed. A detailed breakdown of isolates by species, year, and clinical source is provided in Supplementary Table 1.

Genetic material was extracted using the MagCore Plus II system (RBC Bioscience, New Taipei City, Taiwan) and quantified with a Qubit Flex fluorometer. Sequencing libraries were prepared with the DNA prep kit and Nextera UD indexes (Illumina, San Diego, USA), and sequencing was performed on an Illumina MiSeq platform with 2 × 300 bp read length. Accession numbers of all isolates were included in Genbank Bioproject PRJNA1265187 [Supplementary Table 1].

### 2.4 Data analysis

#### 2.4.1 Statistical and environmental data processing

Results are expressed as percentages calculated by dividing the number of positive cases by total reports (means ± standard deviations). Associations between positive results and the explanatory variables were analyzed using Pearson's chi-square test and Spearman test when necessary. Values with *p* < 0.05 were considered statistically significant. Monthly average SST data from 2010 to 2023 were obtained, and bivariate correlation with clinical cases was analyzed using Spearman's rho test. Values with *p* < 0.05 were considered statistically significant and R values near 1 were considered a strong correlation.

Environmental suitability for *Vibrio* was estimated using an SST threshold >18 °C and air temperature > 22 °C for at least 4 h, as a proxy for recreational water use [9]. Air temperature data were provided by the Naval Station Rota weather station. Statistical analyses were performed using SPSS 25.0.

#### 2.4.2 Genomic analysis

Illumina reads were trimmed and analyzed for quality control. Genome assembly was performed using Shovill's Faster SPAdes. A total of 59 genomic assemblies from Cádiz were annotated using Prokka [20] to obtain standardized gene predictions and functional annotations. The annotated genomes were subsequently analyzed with OrthoFinder [21], which employs the STAG (Species Tree from All Genes) algorithm [22] to infer the unrooted species tree from all gene trees, and the STRIDE (Species Tree Root Inference from Duplication Events) algorithm [23] to determine the most likely root based on gene duplication patterns. This approach enables the reconstruction of a robust inter-species phylogenetic tree, providing an overview of the phylogenetic relationships among the sampled taxa.

To achieve higher phylogenetic resolution at the intra-species level, the Snippy pipeline [24] was executed independently for each species to perform a core genome alignment, from which core single nucleotide polymorphisms (SNPs) were extracted. Based on these core SNP alignments, maximum-likelihood phylogenetic trees were reconstructed using IQ-TREE with 10,000 ultrafast bootstrap replicates to assess branch support [25,26]. The resulting trees were then midpoint-rooted and visualized using iTOL (Interactive Tree Of Life) for graphical representation and comparative interpretation [27].

Sequence types (ST) for *V. parahaemolyticus* and *V. vulnificus* were identified using pubMLST, while Pathogen Watch was used for other species. Gene identification for virulence and antimicrobial resistance was performed using Virulence Factor Database (VFDB, http://www.mgc.ac.cn/VFs/) and the Comprehensive Antibiotic Resistance Database (CARD, http://www.mgc.ac.cn/VFs/).

## 3 Results

### 3.1 Clinical cases and environmental data

A total of 176 isolates from 167 cases of *Vibrio* spp. and related genera infections were identified among the clinical records at hospitals in the region between 2010 and 2023. The most frequently isolated species were *V. alginolyticus* (61.4 %), *S. putrefaciens* (12.5 %), *S. algae* (6.8 %), *V. parahaemolyticus* (6.8 %), *V. fluvialis* (5.7 %), and *V. vulnificus* (2.3 %). Non-toxigenic *V. cholerae* and other *Vibrio* species were identified in less than 2.0 % of the total cases. Coinfections of more than one species were detected in seven patients [Table 1].

**Table 1:** Summary of epidemiological variables (age, sex, species isolated, quarter in the year isolation, and comorbidities).

Category	total	Sepsis, *N* = 7	GII, *N* = 25	Wounds, *N* = 16	Ear, *N* = 105	RTI, N = 7	GUI, *N* = 3	Others*, *N* = 4	X^2^
Age	167								
Median years (range)		60 (48–87)	69 (1–88)	69 (24–90)	36 (3–74)	66 (48–88)	40 (34–44)	55 (9–69)	*p* = 0.26
Sex	167								
M	94	5	10	12	57	7	1	2	*p* = 0.75
F	73	2	15	4	48	0	2	2	
Species	176								
n-t^†^*V*^‡^*. cholerae*	3	1	1	0	1	0	0	0	p < 0.001
*V. parahaemolyticus*	12	1	3	0	7	1	0	0	
*V. vulnificus*	4	1	0	0	3	0	0	0	
*V. alginolyticus*	108	2	7	8	85	2	2	2	
*V. fluvialis*	10	1	9	0	0	0	0	0	
*S*^§^*. algae*	12	2	5	2	3	0	0	0	
*S. putrefaciens*	22	0	1	5	9	4	1	2	
Others^¶^	5	1	0	3	1	0	0	0	
Quarter	167								
First	7	0	1	0	5	0	0	1	*p* = 0.42
Second	24	1	3	5	14	0	1	0	
Third	102	5	17	8	62	5	2	3	
Fourth	34	0	5	3	24	2	0	0	
Comorbidities	71								
Chronic diseases	64	4	15	11	29	3	0	2	*p* = 0.002
Inflammatory diseases	7	0	1	2	2	0	0	2	p < 0.001

*Synovial fluid, conjunctival exudate, and prosthesis. GII, gastrointestinal infections; RTI, respiratory tract infectios; GUI, genitourinary infections. †Non-toxigenic, ‡Vibrio, §Shewanella, ¶*V. harveyi* (1), *V. furnissi* (1), *P. damselae* (1) and *Vibrio* spp. (2).

Most of the clinical cases were associated with otitis (62.9 %), followed by gastroenteritis (15.0 %), infected wounds and ulcers (9.6 %), sepsis (4.2 %), while other less frequent conditions were respiratory, conjunctival, and prosthesis isolates. Five patients showed recurrent infections, with otitis (4/5) and prosthesis infection (1/5), that vary from fortnight to more than a year between medical consultations. Two patients, with wounds and respiratory infections, concurred in systemic progression of the diseases.

Ear infections were caused by several *Vibrio* species, with *V. alginolyticus* (78.0 %) being the most common causative agent, followed by *V. parahaemolyticus* (6.4 %) and *V. vulnificus* (2.8 %). Notably, *V. alginolyticus* was also implicated in cases of gastroenteritis (26.9 %) and wound infections (44.4 %). In contrast, respiratory infections were predominantly associated with *S. putrefaciens* (57.1 %), while *S. algae* was more prevalent in gastroenteritis cases (19.2 %). Interestingly, *V. fluvialis* infections were limited to gastroenteritis (34.6 %) and sepsis (11.1 %). A statistically significant association was detected between the type of clinical affection and the identified species (*p* < 0.001).

Results showed a slight difference between sexes, with 56.3 % males and 43.7 % females and an overall median age of 48 years old (range of 1 to 90). However, it could be observed that patients affected especially by wound infections and sepsis were mainly middle-aged individuals; notably, 63 % of these patients had comorbidities, highlighting the prevalence and impact of these conditions. Overall, the study revealed that 38.9 % of the patients exhibited a chronic and/or auto-inflammatory condition, with a higher prevalence among males (61.5 %) compared to females (38.5 %).

**Fig. 1:**
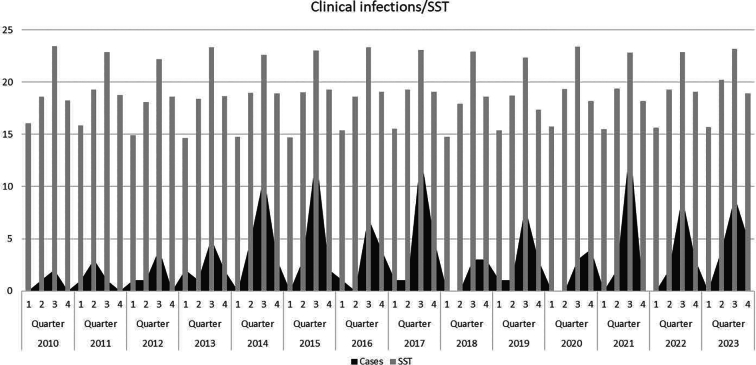
Correlation between number of cases detected in Cádiz (southern Spain) distributed over the study period (2010−2023) divided into quarters and Sea Surface Temperature (SST).

Analyzing the trend of cases over the years, results showed an increase in incidence during the period of study, ranging from 4 cases in 2010 to 17 in 2023 [Fig. 1]. It could also be observed differences in cases occurring between quarters, showing more cases in the third quarter (61.1 %). Interestingly, a shift in the epidemiological pattern was observed over the study period, with cases not reported in the first quarter in the first years and an increasing trend of cases in the fourth quarter over the whole period.

**Fig. 2:**
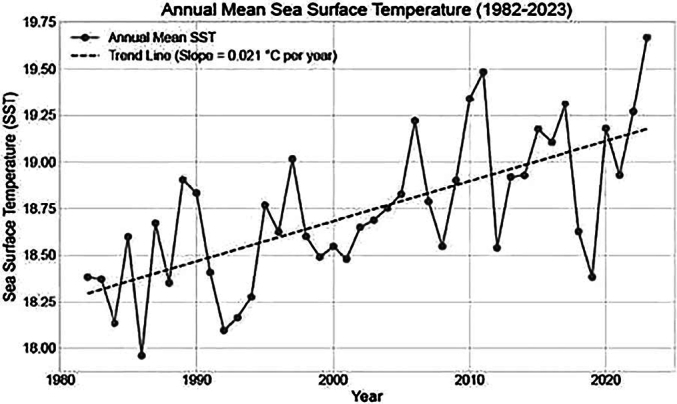

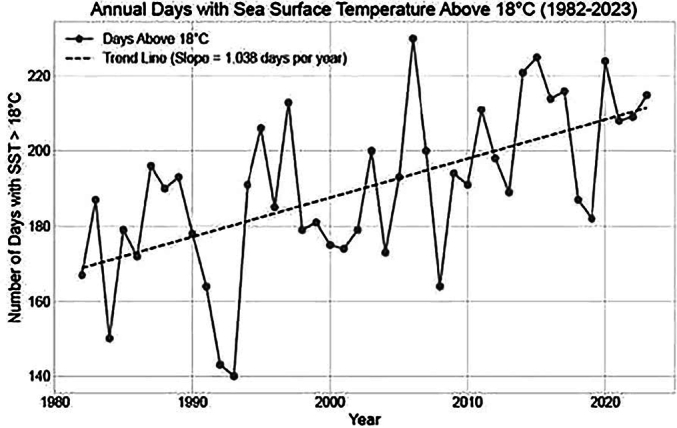

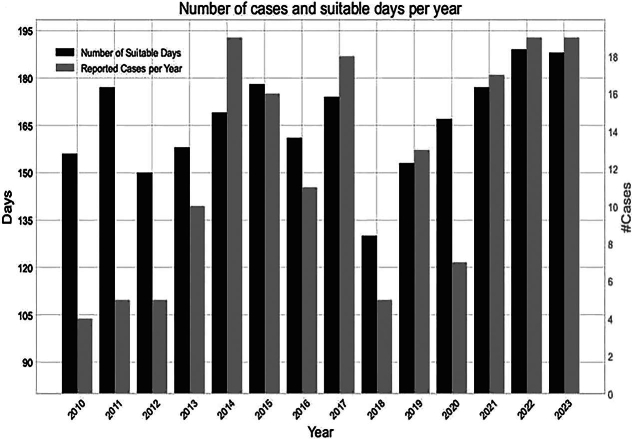
A Annual mean Sea Surface Temperature (SST). B Number of days per year with Sea Surface Temperature (SST) above 18° C from 1982 to 2023. C Number of clinical cases detected in Cádiz (southern Spain) and suitable days per year in this area (2010–2023).

Data of seawater temperature in the region showed a steady warming trend since 1980 with a mean difference of 7.64 °C ± 0.49 between the temperature of the coldest trimester and the hottest. The number of days with temperatures above 18 degrees increased one day per year over the whole period, reaching an expansion of the period with suitable conditions for *Vibrio* of 40 days in the final year. Association between cases and water mean temperature presented statistically significant results (*p* < 0.0001; *R* = 0.64). The number of clinically isolated cases showed a close correspondence with the number of days with favorable environmental conditions for *Vibrio* [Figs. 2A, B and C].

### 3.2 Genetic diversity and predominant sequence types (STs)

Fifty-nine (35.3 %) whole genomes of the pathogens obtained in this study could be sequenced: *V. alginolyticus* (36), *V. parahaemolyticus* (5), *V. vulnificus* (1), non-toxigenic *V. cholerae* (3), *V. fluvialis* (6), *V. furnissi* (1), *S. algae* (6) and *P. damselae* (1).

**Fig. 3:**
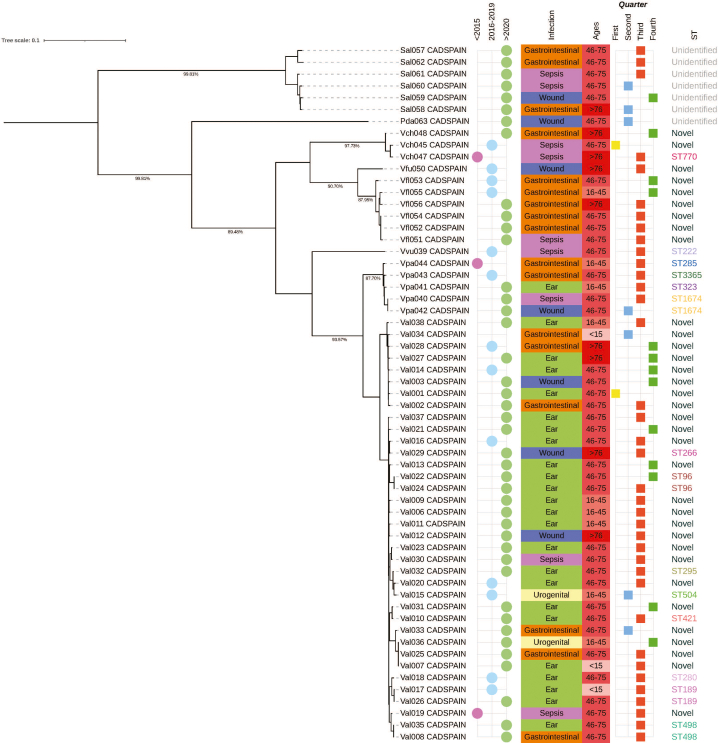
Phylogenetic analysis of *Vibrio, Shewanella*, and *Photobacterium* isolates from southern Spain (2010–2023) (Val, *V. alginolyticus*; Vpa, *V. parahaemolyticus*; Vch, *V. cholerae*; Vfl, *V. fluvialis*, and Sal, *S. algae*).

Our analysis revealed high genetic diversity among 59 *Vibrio* strains. Of the samples with identified sequence types (ST), 32.2 % had known STs, 57.6 % had novel STs, and 10.2 % lacked an identified ST. The most prevalent known STs were ST1674, found in two isolates of *V. parahaemolyticus*, and ST189, ST498, and ST96, which appeared in several isolates of *V. alginolyticus*. To provide an overview of the genetic relatedness among the sequenced isolates, we performed a phylogenetic analysis across *Vibrio*, *Shewanella*, and *Photobacterium* strains. As expected, the analysis primarily reflected species-level separation, serving as an exploratory approach to visualize the diversity present in our dataset rather than to resolve fine-scale phylogenetic relationships [Fig. 3, Supplementary fig. S1-S5].

The remaining *V. parahaemolyticus* genomes were identified as belonging to ST3365, ST285, and ST323, with a single genome. Among the non-toxigenic *V. cholerae* isolates, one was characterized as ST770, with the remaining isolates representing new, previously unidentified STs. Lastly, the *V. vulnificus* isolate was classified as ST222.

### 3.3 Distribution of Virulence and Resistance Genes Among the *Vibrio* Species and related genera

Virulence genes were analyzed in sílico across all genomes [Supplementary fig. S6]. Mannose-sensitive hemagglutinin (MSHA) and type IV pilus genes were found in all species, with lower proportions in *Shewanella* and *Photobacterium*. The *Mam7* adhesion factor was present in all isolates except *Shewanella* and *Photobacterium*.

Regarding secretion systems, the type III secretion system (T3SS1) was only detected in isolates of *V. alginolyticus, V. vulnificus* and *V. parahaemolyticus*. In contrast, two or more T3SS2 effectors was found in very low proportion among the isolates, being detected in some strains of *V. alginolyticus* (8) and specifically *vopA*, *vcrD2*, *vscC2* and *vscN2* in toxigenic isolates of *V. parahaemolyticus* (2) and other species in lower proportion. Finally, VAS type VI secretion system genes were identified in isolates of *V. cholerae*, *V. fluvialis*, *V. furnissi*, *S. algae* and *P. damselae*. Specific toxins like *ace*, *zot*, and *ctxA/B* from *V. cholerae* were absent, while *V. vulnificus* haemolysin *vvhA* was identified. The transcriptional regulator *toxR* was detected in all *V. alginolyticus* and *V. parahaemolyticus* isolates. RTX family toxins were detected in select isolates of *V. cholerae,* and *S. algae*. Two *V. parahaemolyticus* isolates exhibited the presence of the thermostable direct hemolysin (TDH) toxin, as well as TDH-related hemolysin (TRH), in addition to the *hlyA* and *ure* genes, which were also identified in *S. algae* and *P. damselae*. All *V. parahaemolyticus* isolates tested positive for the *tlh* and *toxR* genes.

The CARD database and its Resistance Gene Identifier tool confirmed 24 resistance genes in the genomes [Supplementary fig. S7]. The *TxR* gene, linked to ABC efflux pumps and tetracycline resistance, was found in vibrios of the *Harveyi* group, including *V. alginolyticus* (25/35) and *V. parahaemolyticus* (5/5). The *CRP* gene, associated with RND efflux pumps, was present in most isolates, while the *rsmA* gene was found in *Shewanella* and two *V. parahaemolyticus* isolates.

*β*-lactamase genes were found in all five *V. parahaemolyticus* isolates (two *bla-CARB-18*, two *bla-CARB-20*, and one *bla-CARB-21*), while *bla-CARB-42* was present in all *V. alginolyticus* isolates.

*OXA-55* and *OXA-729* (*OXA-55*-like) carbapenemases were found exclusively in *S. algae*. Mutations in the quinolone target gene *parE* were detected in *Vibrio* but not in *Shewanella* or *Photobacterium*. The *almEFG* operon, linked to colistin resistance in *V. cholerae*, was found in three non-toxigenic *V. cholerae* isolates. Plasmid-encoded quinolone resistance genes *qnrA3* and *qnrA7* were identified in *S. algae*, while the sulfonamide resistance gene *sul2* was detected in three *V. alginolyticus* isolates.

## 4 Discussion

This study provides a comprehensive overview of *Vibrio* and related genera infections along the Atlantic coast of southern Spain over a 13-year period. The findings underscore the diversity of species, clinical impacts, and rising public health significance in the region. As in other European studies, ear and wound infections were most common, followed by gastroenteritis, sepsis, and respiratory infections, with minor regional variations. The predominant pathogen, *V. alginolyticus*, mainly caused otitis, likely due to its biofilm formation and antibiotic resistance, contributing to recurrent infections [12]. Among the non-cholera vibrios*, V. parahaemolyticus* and *V. vulnificus* are the main cause of outbreaks throughout the world, with serious courses that in some cases are fatal. This study linked *V. parahaemolyticus* to ear infections, bacteremia, and respiratory infections in chronically ill patients, showcasing its capacity to affect immunocompromised individuals beyond its traditional association with gastroenteritis [28]. Additionally, *V. vulnificus* was identified in a case of sepsis, underscoring its high mortality risk, particularly among immunocompromised individuals [29]. *S. algae*, noted for its virulence, was associated with a range of infections linked to chronic disease and immunosuppression [30], while the increasing identification of *S. algae* over *S. putrefaciens* suggests the introduction of more accurate microbial identification systems, such as mass spectrometry (MALDI-TOF), could influence the identification of these species [31]. In our study, 21.5 % of patients had hepatic lesions or alcoholism, 71.4 % of whom were men. *Vibrio* infections affect all ages but vary by age: younger individuals had more gastroenteritis and ear infections, while older adults, especially with underlying conditions, experienced respiratory infections and sepsis. *V. vulnificus* infections are most severe in those aged 40–60, representing 50 % of cases and fatalities [29]. An aspect that could not be covered in our study is the availability of detailed epidemiological information regarding patient exposures, such as recreational seawater contact, seafood consumption, or travel history. These limitations direct linkage of clinical manifestations to specific transmission routes. Therefore, our findings should be interpreted as a retrospective descriptive analysis of *Vibrio* and related infections in southern Spain, rather than a full epidemiological investigation. Future prospective studies are warranted to establish exposure–disease relationships.

This study detected a wide variety of STs, showing significant genetic diversity. Some, like ST1674 in *V. parahaemolyticus* and ST222 in *V. vulnificus*, are linked to pathogenic infections [32,33], while 67.8 % of isolates represented new STs, typical of unexplored regions. The observed ST diversity highlights the genetic variability and potential pathogenic risks of *Vibrio* strains, emphasizing the need for ongoing surveillance of emerging threats. The phylogenetic analysis based on a genome-wide-genome approach was performed to provide an overview of the genetic relatedness among *Vibrio*, *Shewanella*, and *Photobacterium* isolates. As expected, isolates are clustered primarily by species, which offers limited novelty and reflects the constraints of applying this methodology across diverse genera. The apparent genetic homogeneity observed among *V. alginolyticus* isolates may reflect either the circulation of closely related clones in the study region or a lack of sensitivity of the pan-genome method to detect fine-scale divergence. More discriminatory approaches, such as core-genome MLST or SNP-based analyses within species, will be required in future studies to assess intra-species diversity more accurately.

Vibriosis incidence in Spain is currently low but likely underestimated due to unreported cases. As shown in this study, retrospective investigations in areas of potential areas risk are surfacing unknown data and providing a different view of these pathogens. Similarly, implementation of monitoring programs in other regions of Spain, such as Galicia (northwest of Spain), had previously identified this region as a hotspot for *Vibrio* spp. disease [34].

The rising prevalence of *Vibrio* infections aligns with climate-driven environmental changes [35]. The Atlantic coast of Cádiz is a popular leisure destination, with a significant increase in population during summer months. The study area, influenced by the Strait of Gibraltar's currents, moderate temperature, and nutrient-rich waters, is a hotspot for *Vibrio* presence and growth. In fact, most *Vibrio* and *Shewanella* infections observed in our study (61.1 %) occurred during the summer months. Because of climate change, global mean SSTs remained persistently and unusually high, reaching record levels for the April–December time of year. This warming of the Atlantic Ocean is primarily driven by thermohaline circulation, which is predominantly influenced by temperature and salinity. Additionally, to these factors, El Niño phenomenon has also been described as having a significant relationship with the dynamics and spread of *Vibrio* infections, especially *V. cholerae* and *V. parahaemolyticus* [36].

Although our data indicates a statistically significant association between seawater warming and *Vibrio* infections, we acknowledge that year-to-year variations do not always show a direct correlation. For instance, years with relatively high SSTs but few clinical cases (2010−2011), as well as 2021 with comparatively moderate SSTs yet higher case numbers, illustrate the contribution of additional factors beyond temperature. These likely include fluctuations in salinity due to freshwater inflows, seasonal extreme weather events, and changes in human exposure or diagnostic practices. Therefore, our findings should be interpreted as evidence of an overall association with warming trends rather than a direct linear correlation across individual years.

Advanced systems enable the prediction of environmental conditions favorable for *Vibrio* growth in coastal waters, utilizing satellite monitoring of sea surface temperatures and salinity to assess risk levels. For example, since July 2014, the ECDC's *Vibrio* Map Viewer has identified highly suitable conditions for *Vibrio* infections. This coincided with a historic peak in vibriosis cases reported by the Baltic Sea region and Sweden's mandatory reporting system in 2014 [37]. By leveraging these predictive tools, healthcare professionals and at-risk individuals can receive timely alerts and proactive guidance to mitigate health risks effectively.

Genomic analysis revealed insights into virulence factors and resistance mechanisms. The analysis of secretion systems in *Vibrio* species and related bacteria reveals significant differences in their distribution and prevalence. The observed predominant presence of T3SS1 secretion system over T3SS2 indicates that cytotoxicity predominates rather than enterotoxicity among our isolates [38,39]. There were also differences in the proportion of type VI secretion system (T6SS) genes between isolates, that suggests that the T3SS1 and 2 systems may be more important for the pathogenesis of *V. alginolyticus, V. vulnificus,* and *V. parahaemolyticus*, while the T6SS may be more relevant for the interactions of *V. cholerae, V. fluvialis, V. furnissii, S. algae,* and *P. damselae*.

The detection of antimicrobial resistance genes indicates a potential risk for emerging resistance. Although no novel genomic profiles were identified in our analysis, it is noteworthy that genes associated with the expression of ABC efflux pumps, commonly found in the *harveyi* group [39], and RND efflux systems, such as *rsmA* in *S. algae* [40], were observed. Furthermore, the presence of *bla-CARB-18*, *bla-CARB-20*, and *bla-CARB-21* genes in *V. parahaemolyticus* isolates is significant. These *β*-lactamase enzymes demonstrate remarkable adaptability, evolving resistance to *β*-lactamase inhibitors through accumulated mutations in amino acid residues.

A key limitation of our study is the underdiagnosis of cases, largely due to the self-limiting nature of these infections, which often leads patients to seek care in primary settings where cultures are not routinely performed. This issue has been widely documented globally, with the US-CDC estimating underreporting rates in the USA as high as 143-fold [41], and a similar scenario is likely for this region. Additionally, the inability to recover all isolates limited the completeness of our clinical strain analyses. Variability in case-patient data further introduced occasional gaps, particularly regarding age and susceptibility profiles. It has also to be noted that improvements in clinical diagnostics and reporting practices over the study period may have contributed to a better identification of *Vibrio* infections, particularly following the introduction of MALDI-TOF technology in 2014. In the absence of a structured surveillance system for vibriosis in Spain, consistent reporting is unlikely. Nevertheless, the long-term trend observed and its strong correlation with warming coastal waters suggest that environmental change is a key underlying driver of the rising number of cases. Future prospective studies incorporating environmental, demographic, and behavioral data (seasonal fluctuations in leisure population, beach attendance, or recreational water use) will be needed to disentangle these overlapping drivers, as these factors may independently increase exposure opportunities and thus contribute to infection risk.

Our study provides new insights into the epidemiological landscape of *Vibrio* infections in southern Spain, a region previously considered of low risk for such illnesses. The retrospective analysis of cases from hospitals has enabled to surface the existence of an epidemiological pattern of *Vibrio* infections which had remained undisclosed with infections primarily linked to recreational water use and, to a lesser extent, seafood consumption. According to the current trends, climate change is expected to expand the environmental suitability for *Vibrio*, but our findings should be interpreted as evidence of an association between warming waters and infection risk, rather than a direct causal relationship. Multiple drivers, including environmental, demographic, and behavioral factors, likely interact to shape the epidemiological landscape. Establishing surveillance systems that integrate clinical, environmental, and population data will be essential to disentangle these contributions and to design effective prevention strategies.

## CRediT authorship contribution statement

**Sandra Tapia-Poza:** Writing – original draft, Software, Investigation, Formal analysis, Data curation. **Estefanía Jurado-Tarifa:** Writing – review & editing, Writing – original draft, Visualization, Software, Investigation, Formal analysis, Data curation, Conceptualization. **Inmaculada Guerrero-Lozano:** Writing – original draft, Methodology, Investigation, Formal analysis, Data curation. **Teresa Trujillo-Soto:** Writing – original draft, Methodology, Formal analysis, Data curation. **Fátima Galán-Sánchez:** Methodology, Investigation, Formal analysis. **Juan M. Sánchez-Calvo:** Investigation, Formal analysis, Data curation. **Joaquín A. Triñanes-Fernández:** Writing – review & editing, Methodology, Investigation, Formal analysis, Data curation. **Jaime Martínez-Urtaza:** Writing – review & editing, Writing – original draft, Validation, Supervision, Resources, Methodology, Investigation, Funding acquisition, Formal analysis, Data curation, Conceptualization. **Manuel A. Rodríguez-Iglesias:** Writing – review & editing, Writing – original draft, Validation, Supervision, Project administration, Methodology, Investigation, Funding acquisition, Formal analysis, Data curation, Conceptualization.

## Ethical statement

The study was reviewed and approved by the Ethics and Research Committee of Cádiz and written informed consent was waived because of the retrospective design.

## Author statement

The corresponding author is responsible for ensuring that the descriptions are accurate and agreed by all authors.

## Funding sources

This study was financially supported by grants for the Financing of Biomedical and Health Sciences Research and Innovation within the Framework of the Integrated Territorial Initiative 2014–2020 for the Province of Cadiz, ITI-FEDER Funds (PI-22-2019). J.M.U. was supported by the 10.13039/501100004837Spanish Ministry of Science and Innovation (PID2021-127107NB-I00 and PID2024-159955NB-100), the 10.13039/501100002809Generalitat de Catalunya (2021 SGR 00526), and the European Union's Horizon Europe research and innovation program (Grant Agreement No. 101057554, IDAlert).

## Declaration of competing interest

The authors declare that they have no known competing financial interests or personal relationships that could have appeared to influence the work reported in this paper.

## Data Availability

The data that has been used is confidential.

## Appendix A Supplementary data

Supplementary material 1 Image 1

Supplementary material 2 Image 2

Supplementary material 3 Image 3
