# Supplementary material for: Emergence of Vibrio and related genera infections in a hotspot of climate risks, southern Spain, 2010–2023
Source: One Health. 2025 Nov 6;21:101267. doi: 10.1016/j.onehlt.2025.101267 (PMC12657601; doi:10.1016/j.onehlt.2025.101267)

Supplementary figure S6. Heatmap of the distribution of virulence factors among *Vibrio* and related genera species.

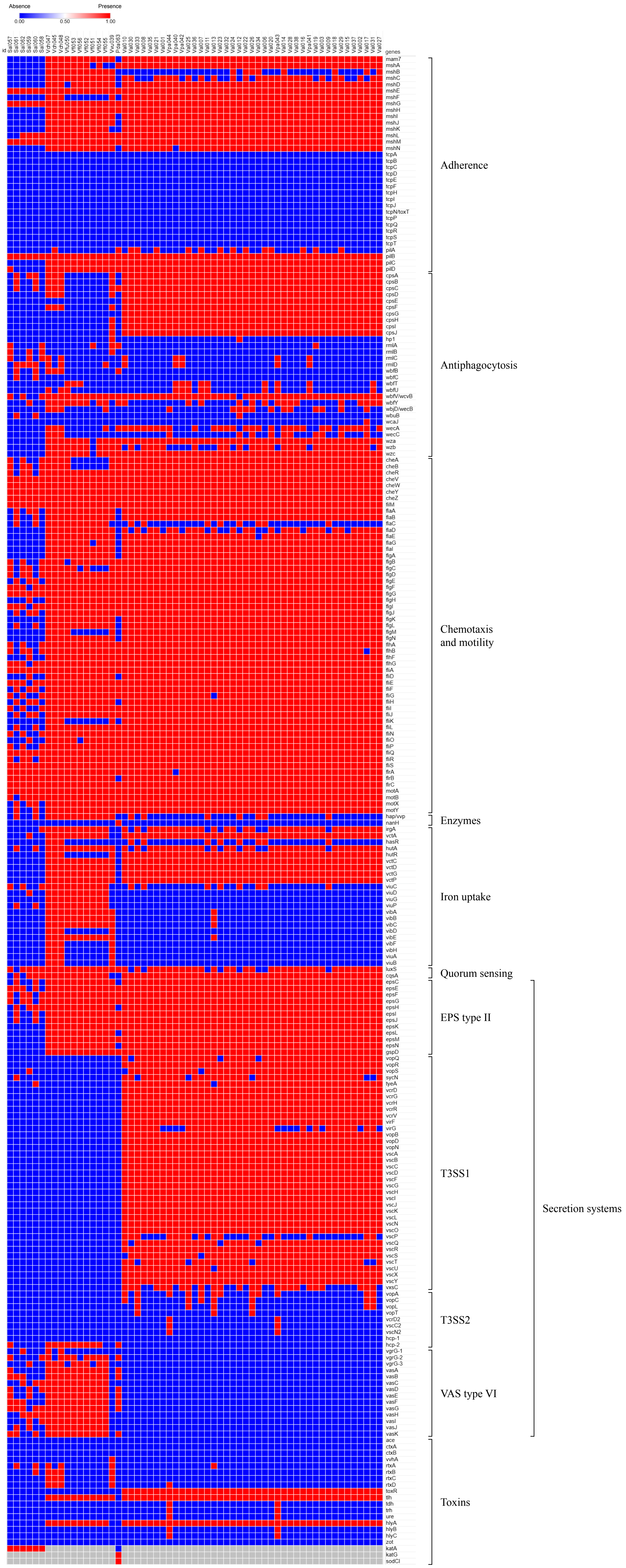

Supplementary figure S7. Heatmap of the distribution of resistance factors among *Vibrio* and related genera species.

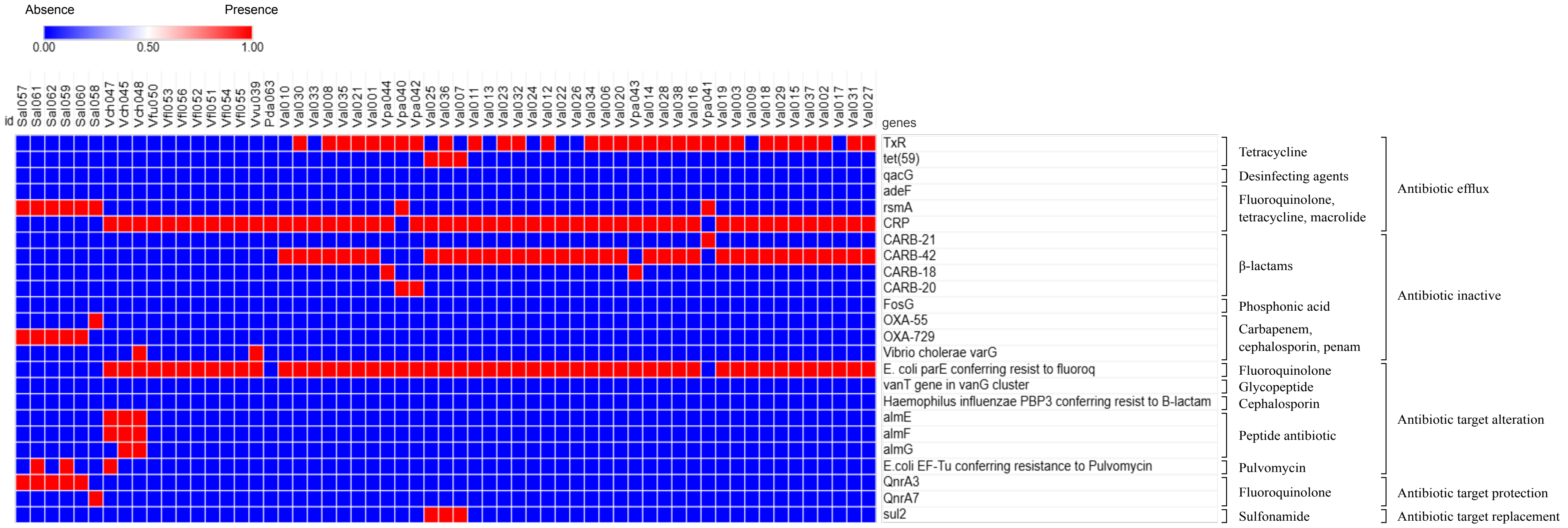

Supplement: Supplementary file 1 — Supplementary material 1 [file mmc1.pdf]
