# Supplementary material for: Emergence of Vibrio and related genera infections in a hotspot of climate risks, southern Spain, 2010–2023
Source: One Health. 2025 Nov 6;21:101267. doi: 10.1016/j.onehlt.2025.101267 (PMC12657601; doi:10.1016/j.onehlt.2025.101267)
Supplement: Supplementary file 2 — Supplementary material 2 [file mmc2.docx]

Supplementary figures S1-S5. Phylogenetic analysis (Snippy pipeline) of *Vibrio parahaemolyticus, Vibrio alginolyticus, Vibrio cholerae, Vibrio fluvialis* and *Shewanella algae*, and Photobacterium isolates from southern Spain (2010–2023).

**S3. *Vibrio parahaemolyticus***

| 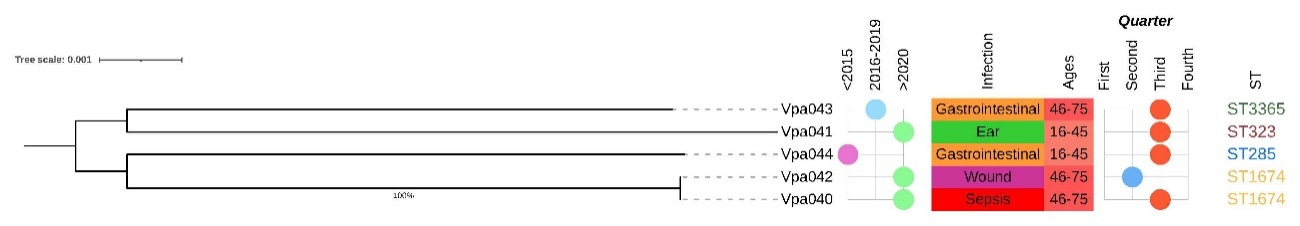 |
| --- |

**S4. *Vibrio alginolyticus***

| 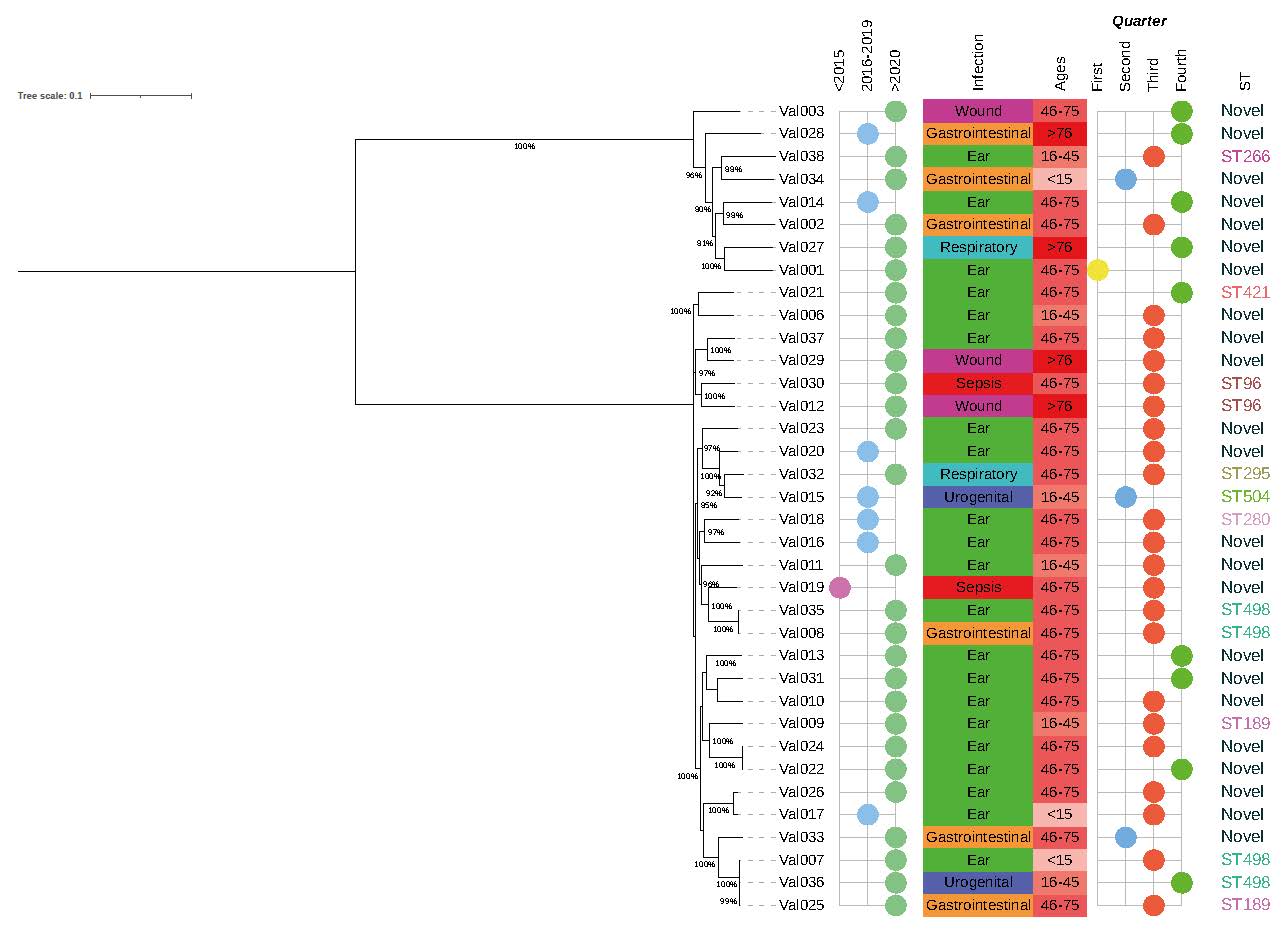 |
| --- |

**S5. *Vibrio cholerae***

| 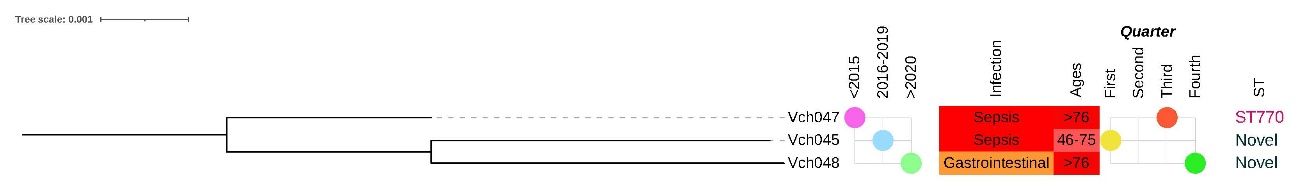 |
| --- |

**S6. *Vibrio fluvialis***

| 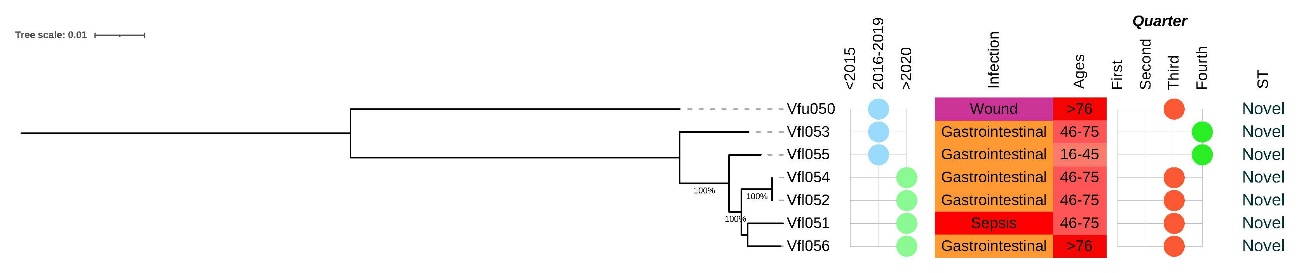 |
| --- |

**S7*. Shewanella algae***

| 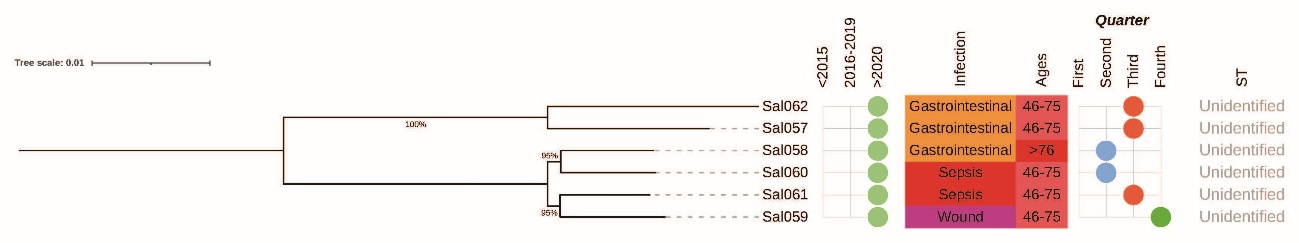 |
| --- |
