# Supplementary material for: Emergence of Vibrio and related genera infections in a hotspot of climate risks, southern Spain, 2010–2023
Source: One Health. 2025 Nov 6;21:101267. doi: 10.1016/j.onehlt.2025.101267 (PMC12657601; doi:10.1016/j.onehlt.2025.101267)
Supplement: Supplementary file 3 — Supplementary material 3 [file mmc3.docx]

**Table S1. Accession numbers in Genbank (Bioproject PRJNA1265187), isolation year and source of 59 *Vibrio* and other genera related**

| Specie | Accession | Sample Name | SPUID | Year | Source |
| --- | --- | --- | --- | --- | --- |
| *Vibrio alginolyticus* | SAMN48586879 | CLINVIBCADSPAIN_Val010 | CLINVIBCADSPAIN_Val010 | 2020 | Ear |
| *Vibrio alginolyticus* | SAMN48586880 | CLINVIBCADSPAIN_Val021 | CLINVIBCADSPAIN_Val021 | 2018 | Ear |
| *Vibrio alginolyticus* | SAMN48588012 | CLINVIBCADSPAIN_Val035 | CLINVIBCADSPAIN_Val035 | 2021 | Ear |
| *Vibrio alginolyticus* | SAMN48588013 | CLINVIBCADSPAIN_Val008 | CLINVIBCADSPAIN_Val008 | 2021 | Gastrointestinal |
| *Vibrio alginolyticus* | SAMN48588014 | CLINVIBCADSPAIN_Val033 | CLINVIBCADSPAIN_Val033 | 2023 | Gastrointestinal |
| *Vibrio alginolyticus* | SAMN48588015 | CLINVIBCADSPAIN_Val030 | CLINVIBCADSPAIN_Val030 | 2021 | Wound |
| *Vibrio alginolyticus* | SAMN48588016 | CLINVIBCADSPAIN_Val001 | CLINVIBCADSPAIN_Val001 | 2019 | Ear |
| *Vibrio alginolyticus* | SAMN48588017 | CLINVIBCADSPAIN_Val025 | CLINVIBCADSPAIN_Val025 | 2021 | Gastrointestinal |
| *Vibrio alginolyticus* | SAMN48588018 | CLINVIBCADSPAIN_Val036 | CLINVIBCADSPAIN_Val036 | 2021 | Urogenital |
| *Vibrio alginolyticus* | SAMN48588019 | CLINVIBCADSPAIN_Val007 | CLINVIBCADSPAIN_Val007 | 2021 | Ear |
| *Vibrio alginolyticus* | SAMN48588020 | CLINVIBCADSPAIN_Val003 | CLINVIBCADSPAIN_Val003 | 2020 | Wound |
| *Vibrio alginolyticus* | SAMN48588021 | CLINVIBCADSPAIN_Val012 | CLINVIBCADSPAIN_Val012 | 2019 | Wound |
| *Vibrio alginolyticus* | SAMN48588022 | CLINVIBCADSPAIN_Val031 | CLINVIBCADSPAIN_Val031 | 2020 | Ear |
| *Vibrio alginolyticus* | SAMN48588023 | CLINVIBCADSPAIN_Val034 | CLINVIBCADSPAIN_Val034 | 2023 | Gastrointestinal |
| *Vibrio alginolyticus* | SAMN48588024 | CLINVIBCADSPAIN_Val024 | CLINVIBCADSPAIN_Val024 | 2023 | Ear |
| *Vibrio alginolyticus* | SAMN48588025 | CLINVIBCADSPAIN_Val016 | CLINVIBCADSPAIN_Val016 | 2029 | Ear |
| *Vibrio alginolyticus* | SAMN48588026 | CLINVIBCADSPAIN_Val037 | CLINVIBCADSPAIN_Val037 | 2021 | Ear |
| *Vibrio alginolyticus* | SAMN48588027 | CLINVIBCADSPAIN_Val013 | CLINVIBCADSPAIN_Val013 | 2019 | Ear |
| *Vibrio alginolyticus* | SAMN48588028 | CLINVIBCADSPAIN_Val015 | CLINVIBCADSPAIN_Val015 | 2019 | Urogenital |
| *Vibrio alginolyticus* | SAMN48588029 | CLINVIBCADSPAIN_Val029 | CLINVIBCADSPAIN_Val029 | 2021 | Wound |
| *Vibrio alginolyticus* | SAMN48588030 | CLINVIBCADSPAIN_Val018 | CLINVIBCADSPAIN_Val018 | 2017 | Ear |
| *Vibrio alginolyticus* | SAMN48588031 | CLINVIBCADSPAIN_Val028 | CLINVIBCADSPAIN_Val028 | 2022 | Gastrointestinal |
| *Vibrio alginolyticus* | SAMN48588238 | CLINVIBCADSPAIN_Val038 | CLINVIBCADSPAIN_Val038 | 2021 | Ear |
| *Vibrio alginolyticus* | SAMN48588239 | CLINVIBCADSPAIN_Val011 | CLINVIBCADSPAIN_Val011 | 2019 | Ear |
| *Vibrio alginolyticus* | SAMN48588240 | CLINVIBCADSPAIN_Val027 | CLINVIBCADSPAIN_Val027 | 2022 | Ear |
| *Vibrio alginolyticus* | SAMN48588241 | CLINVIBCADSPAIN_Val020 | CLINVIBCADSPAIN_Val020 | 2017 | Ear |
| *Vibrio alginolyticus* | SAMN48588242 | CLINVIBCADSPAIN_Val014 | CLINVIBCADSPAIN_Val014 | 2019 | Ear |
| *Vibrio alginolyticus* | SAMN48588243 | CLINVIBCADSPAIN_Val032 | CLINVIBCADSPAIN_Val032 | 2018 | Ear |
| *Vibrio alginolyticus* | SAMN48588244 | CLINVIBCADSPAIN_Val017 | CLINVIBCADSPAIN_Val017 | 2019 | Ear |
| *Vibrio alginolyticus* | SAMN48588245 | CLINVIBCADSPAIN_Val023 | CLINVIBCADSPAIN_Val023 | 2023 | Sepsis |
| *Vibrio alginolyticus* | SAMN48588246 | CLINVIBCADSPAIN_Val009 | CLINVIBCADSPAIN_Val009 | 2023 | Ear |
| *Vibrio alginolyticus* | SAMN48588247 | CLINVIBCADSPAIN_Val006 | CLINVIBCADSPAIN_Val006 | 2023 | Ear |
| *Vibrio alginolyticus* | SAMN48588248 | CLINVIBCADSPAIN_Val022 | CLINVIBCADSPAIN_Val022 | 2023 | Ear |
| *Vibrio alginolyticus* | SAMN48588249 | CLINVIBCADSPAIN_Val019 | CLINVIBCADSPAIN_Val019 | 2015 | Ear |
| *Vibrio alginolyticus* | SAMN48588250 | CLINVIBCADSPAIN_Val026 | CLINVIBCADSPAIN_Val026 | 2020 | Ear |
| *Vibrio alginolyticus* | SAMN48588251 | CLINVIBCADSPAIN_Val002 | CLINVIBCADSPAIN_Val002 | 2022 | Gastrointestinal |
| *Vibrio cholerae* | SAMN48586867 | CLINVIBCADSPAIN_Vch047 | CLINVIBCADSPAIN_Vch047 | 2019 | Sepsis |
| *Vibrio cholerae* | SAMN48586868 | CLINVIBCADSPAIN_Vch045 | CLINVIBCADSPAIN_Vch045 | 2016 | Sepsis |
| *Vibrio cholerae* | SAMN48586869 | CLINVIBCADSPAIN_Vch048 | CLINVIBCADSPAIN_Vch048 | 2022 | Gastrointestinal |
| *Vibrio fluvialis* | SAMN48586871 | CLINVIBCADSPAIN_Vfl053 | CLINVIBCADSPAIN_Vfl053 | 2016 | Gastrointestinal |
| *Vibrio fluvialis* | SAMN48586872 | CLINVIBCADSPAIN_Vflu052 | CLINVIBCADSPAIN_Vflu052 | 2023 | Gastrointestinal |
| *Vibrio fluvialis* | SAMN48586873 | CLINVIBCADSPAIN_Vflu054 | CLINVIBCADSPAIN_Vflu054 | 2023 | Gastrointestinal |
| *Vibrio fluvialis* | SAMN48586874 | CLINVIBCADSPAIN_Vflu056 | CLINVIBCADSPAIN_Vflu056 | 2016 | Sepsis |
| *Vibrio fluvialis* | SAMN48586875 | CLINVIBCADSPAIN_Vflu055 | CLINVIBCADSPAIN_Vflu055 | 2020 | Gastrointestinal |
| *Vibrio fluvialis* | SAMN48586876 | CLINVIBCADSPAIN_Vflu051 | CLINVIBCADSPAIN_Vflu051 | 2022 | Gastrointestinal |
| *Vibrio furnissii* | SAMN48586870 | CLINVIBCADSPAIN_Vfu050 | CLINVIBCADSPAIN_Vfu050 | 2019 | Wound |
| *Vibrio parahaemolyticus* | SAMN48588252 | CLINVIBCADSPAIN_Vpa042 | CLINVIBCADSPAIN_Vpa042 | 2021 | Wound |
| *Vibrio parahaemolyticus* | SAMN48588253 | CLINVIBCADSPAIN_Vpa040 | CLINVIBCADSPAIN_Vpa040 | 2022 | Sepsis |
| *Vibrio parahaemolyticus* | SAMN48588254 | CLINVIBCADSPAIN_Vpa044 | CLINVIBCADSPAIN_Vpa044 | 2015 | Gastrointestinal |
| *Vibrio parahaemolyticus* | SAMN48588255 | CLINVIBCADSPAIN_Vpa043 | CLINVIBCADSPAIN_Vpa043 | 2019 | Gastrointestinal |
| *Vibrio parahaemolyticus* | SAMN48588256 | CLINVIBCADSPAIN_Vpa041 | CLINVIBCADSPAIN_Vpa041 | 2021 | Ear |
| *Vibrio vulnificus* | SAMN48586877 | CLINVIBCADSPAIN_Vvu039 | CLINVIBCADSPAIN_Vvu039 | 2017 | Sepsis |
| *Shewanella algae* | SAMN48586861 | CLINVIBCADSPAIN_Sal057 | CLINVIBCADSPAIN_Sal057 | 2021 | Gastrointestinal |
| *Shewanella algae* | SAMN48586862 | CLINVIBCADSPAIN_Sal062 | CLINVIBCADSPAIN_Sal062 | 2021 | Wound |
| *Shewanella algae* | SAMN48586863 | CLINVIBCADSPAIN_Sal061 | CLINVIBCADSPAIN_Sal061 | 2022 | Sepsis |
| *Shewanella algae* | SAMN48586864 | CLINVIBCADSPAIN_Sal058 | CLINVIBCADSPAIN_Sal058 | 2022 | Gastrointestinal |
| *Shewanella algae* | SAMN48586865 | CLINVIBCADSPAIN_Sal060 | CLINVIBCADSPAIN_Sal060 | 2023 | Gastrointestinal |
| *Shewanella algae* | SAMN48586866 | CLINVIBCADSPAIN_Sal059 | CLINVIBCADSPAIN_Sal059 | 2023 | Sepsis |
| *Photobacterium damselae* | SAMN48586878 | CLINVIBCADSPAIN_Pda063 | CLINVIBCADSPAIN_Pda063 | 2021 | Wound |
